# Prognostic value of in vitro growth pattern of colony forming cells in adult acute leukaemia.

**DOI:** 10.1038/bjc.1983.64

**Published:** 1983-03

**Authors:** U. Jehn, D. Kern, K. Wachholz, D. Hölzel


*Br. J. Cancer* (1983), **47**, 423–428

### Short Communication

### Prognostic value of *in vitro* growth pattern of colony forming cells in adult acute leukaemia

U. Jehn, D. Kern, K. Wachholz & D. Hölzel[1]

*Medizinische Klinik III and [1]Institut für Informationsverarbeitung, Statistik, Biomathematik Klinikum Grosshadern—University of Munich.*

A number of recent studies on acute leukaemia have focused on pretreatment factors that are of prognostic significance in determining the likelihood of achieving complete remission, remission duration or length of survival. Even so, it has been difficult to identify convincing clinical or haematological criteria for predicting the response to cytostatic drugs. Determination of colony formation has become a useful tool for investigating the abnormal regulation of haematopoiesis. Therefore, several investigators have tried to correlate the *in vitro* growth pattern of granulopoietic progenitor cells with the prognostic outcome (Keating *et al.*, 1980; Elias & Greenberg, 1977; Moore *et al.*, 1974; Hörnsten *et al.*, 1977; Beran *et al.*, 1980; Gustavsson *et al.*, 1981). However, the results have been conflicting due to poor reproducibility and differences in culture technique, sources of colony stimulating activity, classification of growth pattern, therapeutic regimens, selection of patients, and inadequate statistical analysis. The objective of the present study was (a) to investigate in each patient with acute myelogenous (AML) and lymphocytic leukaemia (ALL), the *in vitro* colony-forming capacity of both bone marrow and peripheral blood at first diagnosis, during induction treatment, in remission and relapse; and (b) to evaluate the prognostic utility of this *in vitro* assay in respect of rate of remission, remission duration, and length of survival.

Fifty consecutive, previously untreated adults with acute leukaemia were investigated over a 2 yr-period; 31 were cases of AML and 19 were of ALL. The morphological and immunological variants as well as clinical details are summarized in Table I. For remission induction, AML-patients were treated with adriamycin i.v. ($50 \, mg \, m^{-2}$) Day 1, vincristine i.v. ($1 \, mg \, m^{-2}$) Day 2, cytosine arabinoside i.v. ($80 \, mg \, m^{-2}$) as push injection every 12 h Days 3–9. Bone marrow was evaluated 15–21 days after the end of each cycle. Two–4 courses were given, including one course of consolidation for patients who achieved complete remission. Treatment was discontinued after the 3rd cycle in patients who failed to respond. For maintenance, the majority of patients received oral thioguanin ($70 \, mg \, m^{-2}$) on 4 consecutive days and cytosine arabinoside subcutaneously ($80 \, mg \, m^{-2}$) on the 5th day, weekly till relapse.

Initial induction treatment for ALL-patients consisted of adriamycin i.v. ($30 \, mg \, m^{-2}$) and vincristine i.v. ($1.4 \, mg \, m^{-2}$) Days 0, 14, 28, 42, prednisolone by mouth (40 mg) daily with or without L-asparaginase i.v. ($10{,}000 \, IU \, m^{-2}$) Days 0–14 followed by either prophylactic or therapeutic cranial irradiation and intrathecal methotrexate. Bone marrow was evaluated before each injection of adriamycin. Remission was maintained with oral mercaptopurine (75 mg daily), oral cyclophosphamide 300 mg/week and oral methotrexate 30 mg/week. Dosages of all drugs were adjusted to keep the total white blood cell count at $3 \times 10^9 \, l^{-1}$. Patients were treated until relapse. Treatment of relapse was in the majority of cases identical to the primary induction therapy.

All patients had blood and bone marrow smears stained by May-Grünwald-Giemsa, PAS, peroxidase, nonspecific esterase with and without sodium fluoride, acid phosphatase with and without tartrate. Complete remission status required the patient to be in normal health with haemoglobin $> 10 \, gm \, dl^{-1}$, granulocytes $> 1.5 \times 10^9 \, l^{-1}$, platelets counts $\geqslant 100 \times 10^9 \, l^{-1}$, and normal bone marrow cellularity with $< 5\%$ blast cells. As a rule, clusters and colonies were studied in both bone marrow and peripheral blood simultaneously at first diagnosis, after each course of induction treatment, at remission, during remission in $\sim 2$ month intervals, and when relapse occurred. Fourteen patients with AML and 8 with ALL were followed until relapse and during their reinduction therapy. Fourteen healthy donors served as normal controls for the *in vitro* growth pattern of $CFU_c$ in the bone marrow.

The double-layer agar technique of Pike &

Received 7 October 1982; accepted November 1982.

Correspondence: U. Jehn Med. Klinik III, Klinikum Grosshadern Marchioninistr. 15 8000 München/Munich 70 F.R.G.

© The Macmillan Press Ltd., 1983.

**Table I** Clinical characteristics of 50 patients investigated. AML-patients are listed according to morphology and cytochemistry. M* refers to the FAB-classification † = one patient with acute mixed leukaemia but myeloid predominance of leukaemic blasts. ALL is listed according to immunological marker analysis. ‡n.d. = not done.

| | | *n* | *age* (*mean*) | *sex* (*m:f*) |
|---|---|---|---|---|
| AML | | | | |
| myeloblastic | (M 1+2*) | 13 | | |
| myelomonocytic | (M 4*) | 12 | | |
| monoblastic | (M 5*) | 4 | 18–79 (50) | 15:16 |
| promyelocytic | (M 3*) | 1 | | |
| mixed† | | 1 | | |
| total | | 31 | | |
| ALL | | | | |
| pre-T | | 4 | | |
| T | | 5 | | |
| c/ALL | | 4 | | |
| c/T | | 1 | | |
| AUL | | 1 | 16–72 (42) | 8:11 |
| Burkitt-type | | 3 | | |
| n.d.‡ | | 1 | | |
| total | | 19 | | |

Robinson (1970) was used with some minor modifications. Feeder layers contained $10^6$ blood mononuclear cells $ml^{-1}$, isolated on Isopaque-Ficoll 1.077 $g ml^{-1}$, and supplemented with 40% of the corresponding granulocytes, both obtained from the same 2 donors throughout the study, in order to eliminate unpredictable variations in the production of colony stimulating activity found with different leukocyte feeder layers. Feeder layers were always prepared in parallel from both donors. Colony formation was assayed using 2 standard, increasing concentrations of patients mononuclear cells: $10^5 ml^{-1}$ and $2 \times 10^5 ml^{-1}$ for bone marrow, $0.5 \times 10^6 ml^{-1}$ and $10^6 ml^{-1}$ for peripheral blood. Cultures were scored with an inverted microscope at ×40 and ×25 magnification for clusters of 3–50 cells or colonies if >50 cells after 10 days. Each determination represents the mean value of several dishes. Clusters and colonies were expressed per ml peripheral blood and bone marrow (Blacket & Gordon, 1979), taking several variables into account such as the differential count, absolute number of leukocytes and the quantity of the specimen investigated. Heparinized bone marrow (1–2 ml) was obtained by aspiration from the anterior or posterior iliac crests. Simultaneously, 20 ml heparinized blood was collected. The morphology of cells within colonies and clusters was not monitored systematically but in a few cases studied cultures contained granulocytic cells up to a metamyelocytic stage.

For the analysis of remission duration and survival, the life-table method of Cutler and Ederer was used. Non-parametric variance analysis according to the H-test of Kruscal and Wallis was employed to examine the differences in single pretreatment variables in therapy responsive (R) and non-responsive (NR) groups of patients as well as normal individuals. When 2 independent samples were compared, the Mann-Whitney test was used. For the analysis of frequencies the $\chi^2$-test or Fisher's exact probability test was used. To analyse the relation of pretreatment characteristics and length of remission or survival, non-parametric correlation coefficient was calculated according to the method of Spearman. The level of significance in all tests was $P \leqq 0.05$.

Clinical findings are summarized in Table II. The patient population was unselected, including antecedent haematological disorders and other malignancies (pre-leukaemic state 2, refractory sideroblastic anaemia 1, haemolytic anaemia 1, aplastic anaemia 1, breast cancer 2, patients older than 65 y (8), and patients with signs of infection (fever >38°C) at admission. There was no significant difference in the rate of remission, or distribution of morphological and growth patterns among these subgroups.

The number of cluster- or colony-forming cells of patients with all forms of acute leukaemia varied over a wide range, particularly in the marrow, at first presentation. Even so, for both AML and ALL,

**Table II** Treatment results of patients with acute leukaemia after induction (at first diagnosis) and re-induction (at relapse) therapy. Median remission duration and median survival was calculated according to life table analysis. R = responder, NR = non-responder, mo = months.

| | *n* | *rate of remission* (%) | *remission duration* (*R in mo*) | *survival* (*R+NR in mo*) |
|---|---|---|---|---|
| AML | | | | |
| at first diagnosis | 31 | 15 (48) | 8 | 9.4 |
| at relapse | 14 | 8 (57) | 3.4 | 10.4 |
| ALL | | | | |
| at first diagnosis | 19 | 14 (74) | 11 | 7.8 |
| at relapse | 8 | 3 (38) | 1 | 2.8 |

colony-growth of the bone marrow was significantly poorer than in normal individuals, while colonies of the peripheral blood remained within the normal range. The number of marrow colonies was lowest after induction treatment, when remission was achieved, in spite of a normal cellularity and blast counts <5%. In AML, the bone marrow pool of colonies recovered 1–2 months after remission occurred, in ALL after 3–4 months. In both types of leukaemia, however, it never regained normal values throughout the observation period of 7–8 months. The growth pattern of the peripheral blood was contrary to that of the bone marrow: the number of colonies continuously decreased and, after 3–4 months were found only sporadically.

At first diagnosis, marrow clusters in AML and ALL were within normal range, and clusters from the peripheral blood were significantly above normal controls. At remission, the number of clusters in the bone marrow was significantly lower than before therapy. In contrast to colony-growth, bone marrow cluster in AML and ALL showed an overshoot 3–4 months after remission occurred and remained at normal levels during remission. Clusters disappeared from the peripheral blood, although 3–4 months later than colonies. The *in vitro* growth pattern of both colonies and clusters in marrow and blood during relapse was similar to that at first diagnosis.

AML-patients responding to therapy showed (Figure 1) less colony-growth and significantly less cluster-growth in bone marrow and displayed more frequent negative cultures than those failing to respond. In contrast, blood cultures (Figure 2) had significantly more colonies and clusters in the responder than non-responder group, the latter containing significantly more negative cultures. In other words, poor *in vitro* growth in the bone marrow and normal or high numbers of $CFU_c$ in the peripheral blood, ensured for AML patients a good chance of reaching complete remission. Comparison of remission incidence in marrow and blood of ALL-patients showed that the growth pattern was opposite to that of patients with AML: more colony and cluster growth in the bone marrow and normal growth in the peripheral blood in patients responding to therapy than in the non-responder group.

In good agreement with this is the observation that patients with AML can be expected to have significantly longer remission ($P \leq 0.02$) when high numbers of clusters and colonies are found in the peripheral blood, before therapy is initiated. No such correlation existed for patients with ALL prior to induction therapy. At relapse, however, patients showing many clusters and/or colonies in the marrow, had significantly longer remissions ($P = 0.03/0.01$) than those with poor bone marrow growth. The pretreatment growth-characteristics were not of predictive significance for survival in either AML or ALL.

The degree of marrow infiltration with blast cells was not of prognostic value. In AML, the mean values were 65% blasts in the therapy-responsive compared with 54% in the non-responsive group. The corresponding figures for ALL patients were 72% and 73%. However in ALL, high blast cell counts in the bone marrow correlated significantly with many clusters in the peripheral blood, indicating a blast cell-dependent mechanism for emigration of normal progenitor cells from marrow into the peripheral blood.

There is strong evidence that clusters and colonies growing under the culture conditions used in this study, are derived from normal residual stem cells (Metcalf & Moore, 1971; Pike & Robinson, 1970) and not from leukaemic cells (Hoelzer *et al.*, 1977). Even so, there seems no simple way of determining the origin of colonies and clusters. In a recent study (Marie *et al.*, 1981), evidence was presented that in AML a minority of clusters are formed from blast cells and some granulopoietic differentiation may be expressed during blast colony formation even though morphological evidence of

**Figure 1** Distribution of clusters (clu) and colonies (col) per ml bone marrow in normal controls and in AML-patients at first diagnosis. Values are listed according to whether complete remission was obtained or not. Responders showed less colonies and significantly less clusters than non-responders ($P \leqslant 0.05$).

differentiation is not apparent. We conclude from the overshooting reappearance of clusters in the bone marrow 1–2 months after haematological remission had been reached and from the continuous disappearance in the peripheral blood during remission, that the clusters seen in our experiments represent normal granulopoietic progenitor cells.

Our observation that clusters found in the bone marrow of both leukaemias during remission were far above normal while bone marrow colonies never return to normal levels, is in good agreement with the finding (Broxmeyer *et al.*, 1979) of persistent inhibitory activity against the formation of granulocyte and macrophage colonies in cultures of normal bone marrow cells during remission of acute leukaemia.

All patients followed during induction treatment showed some reduction in colony-forming ability, regardless of whether they reached complete remission or not. The lowest values were found when patients had recovered from leukopenia and remission was first diagnosed. This suggests that cytotoxic drugs affect $CFU_c$ with some latency (Necas *et al.*, 1979; Francis *et al.*, 1981). Colony-forming capacity reappeared fully ~1 month after remission was attained. A second nadir occurred in association with the antecedent consolidation cycle.

The divergent pattern of blood and marrow culture growth in our patients with AML and ALL

**Figure 2** Distribution of clusters (clu) and colonies (col) per ml peripheral blood in normal controls and at first diagnosis. Values are listed according to whether complete remission was obtained or not. Responses showed significantly more clusters and colonies ($P \leq 0.05$) than non-responders . In addition, the poor-prognosis group showed significantly ($P \leq 0.05$) more negative cultures in the peripheral blood than responders.

in respect of clinical outcome regards some comment. In agreement with a recent study (Beran *et al.*, 1980), AML patients showing an increased number of clones in marrow cultures responded poorly to therapy, but those with a decreased clone number, predominantly clusters, and a high percentage of negative leukaemias achieved significantly more frequent complete remission. In the circulating blood, the situation was opposite to that in bone marrow *viz.* high numbers of colony and clusters forming cells and less negative cultures in the good prognosis group. This relation between a favourable prognosis in terms of remission incidence and the presence of colony-forming cells in the peripheral blood has been noticed earlier (Hörnsten *et al.*, 1977). Furthermore, longer remissions were much more frequent among AML-patients whose blood cells showed many colonies and clusters. In a multivariant analysis of several pretreatment factors, Keating *et al.* (1980) found a similar significant correlation between high numbers of clusters and long remission. Since on the other hand, our non-responding group has less colonies and clusters in the peripheral blood than responders, one possible explanation of our results is that in AML the remaining normal haematopoiesis of those leukaemias which can respond to therapy deserts the homing bone marrow and migrates into the peripheral blood. According to our results such a mechanism would be independent of the degree of blast infiltration. In the poor prognosis group, such an escape of normal granulopoiesis does not occur and would be ultimately rejected.

In the ALL-group, although small in size, the results differed markedly from those found in AML-patients. The *in vitro* growth of blood clusters and colonies remained within normal limits, equally in responsive and non-responsive patients. For ALL-patients prior to any therapy, there was a definite trend (although insignificant) toward achieving remission and obtaining long remissions when high $CFU_c$ counts were found in the marrow culture. Both correlations were significant at relapse. Cell surface characteristics have uncovered a variety of ALL-subgroups with close correlation to the prognosis of the disease (Greaves *et al.*, 1978). At first diagnosis we found 6 different types, but at relapse only 2 remained. This might explain the slight discrepancies in significance between the time of first presentation and relapse. In ALL, no emigration phenomenon with prognostic relevance could be observed. However, there was a significant correlation between blast infiltration of the marrow and the number of clusters in the peripheral blood in that, high numbers of blast cells in the bone marrow implicated high *in vitro* growth of peripheral blood.

Although much of the information obtained relies upon group analysis the conclusion is that, particularly in AML, and to a minor degree in ALL, the *in vitro* growth pattern at diagnosis is a prognostic indicator of whether or not patients will respond to chemotherapy and experience long remissions. A good prognosis in terms of remission rate and duration does not necessarily imply a long survival.

### References

BERAN, M., REIZENSTEIN, P. & UDEN, A.M. (1980). Response to treatment in acute non-lymphatic leukaemia: prognostic value of colony forming and colony stimulating capacities of bone marrow and blood cells compared to other parameters. *Br. J. Haematol.*, **44**, 39.

BLACKET, N.M. & GORDON, M.Y. (1979). Observations on the distribution of granulocytic progenitor cells (CFU-c) in human bone marrow: the importance of the manner in which the results of *in vitro* cultures are reported. *Br. J. Haematol.*, **39**, 353.

BROXMEYER, H.E., GROSSBARD, E., JACOBSEN, N. & MOORE, M.A.S. (1979). Persistence of inhibitory activity against normal bone marrow cells during remission of acute leukaemia. *N. Engl. J. Med.*, **301**, 346.

ELIAS, L. & GREENBERG, P. (1977). Divergent patterns of marrow cells suspension culture growth in the myeloid leukaemias: correlation of *in vitro* findings with clinical findings. *Blood*, **50**, 263.

FRANCIS, G.E., TUNA, G.A., BEREY, J. & HOFFBRAND, A.V. (1981). Sensitivity of acute myeloid leukaemia cells to colony stimulating activity: relation to response to chemotherapy. *Br. J. Haematol.*, **49**, 259.

GORDON, M.Y. & DOUGLAS, I.D.C. (1977). The effect of peripheral blood contamination on colony yield from human bone marrow aspirates. *Exp. Haematol.*, **5**, 574.

GREAVES, M.F., JANOSSY, G., FRANCIS, G.E. & MINOWADA, J. (1978). Membrane phenotypes of human leukaemic cells and leukaemic cell lines. Clinical correlates and biological implications. In *Differentiation of Normal and Neoplastic Hematopoietic Cells*, New York: Cold Spring Harbor Laboratory p. 823.

GUSTAVSSON, A., OLOFSSON, T., OLSSON, I. & SIGURDARDOTTIR, H. (1981). The prognostic significance of *in vitro* bone marrow growth pattern in acute non-lymphocytic leukaemia. *Scand. J. Hematol.*, **26**, 364.

HOELZER, D., KURRLE, E., SCHMÜCKER, H. & HARRISS, E.B. (1977). Evidence for differentiation of human leukaemic blood cells in diffusion chamber culture. *Blood*, **49**, 729.

HÖRNSTEN, P., GRANSTRÖM, M., WAHREN, B. & GAHRTON, G. (1977). Prognostic value of colony-stimulating and colony-forming cells in peripheral blood in acute non-lymphoblastic leukaemia. *Acta Med. Scand.*, **201**, 405.

KEATING, M.I., SMITH, T.L., GEHAN, E.A. & 6 others. (1980). Factors related to length of complete remission in adult acute leukaemia. *Cancer*, **45**, 2017.

MARIE, J.P., IZAGUIRE, A., CIVIN, C.I., MIRRO, J. & MCCULLOCH, A. (1981). Granulopoietic differentiation in AML blasts in culture. *Blood*, **58**, 670.

METCALF, D. & MOORE, M.A.S. (1971). Haemopoietic stem cells and progenitor cells. In *Haemopoietic Cells* Amsterdam: North Holland Publishing Co, P. 70.

MOORE, M.A.S., SPITZER, G., WILLIAMS, N., METCALF, D. & BUCKLEY, J. (1974). Agar culture studies in 127 cases of untreated acute leukaemia: the prognostic value of reclassification of leukaemia according to *in vitro* growth characteristics. *Blood* **44**, 1.

NECAS, E., PONKA, P. & NEUWIRT, J. (1979). Effect of some cytostatics on the haemopoietic stem cells (CFU-c) in blood. *Cancer Chemother. Pharmacol.*, **2**, 215.

PIKE, B.L. & ROBINSON, W.A. (1970). Human bone marrow colony growth in agar gel. *J. Cell. Physiol.*, **76**, 77.


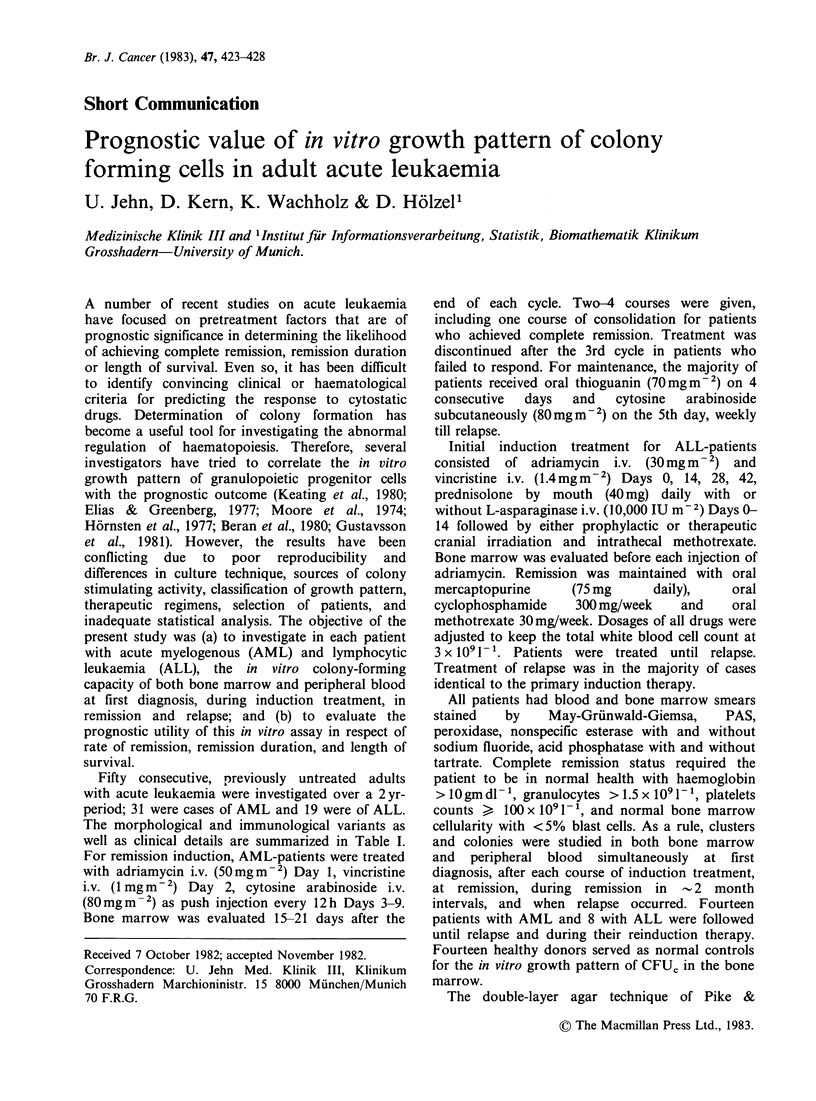

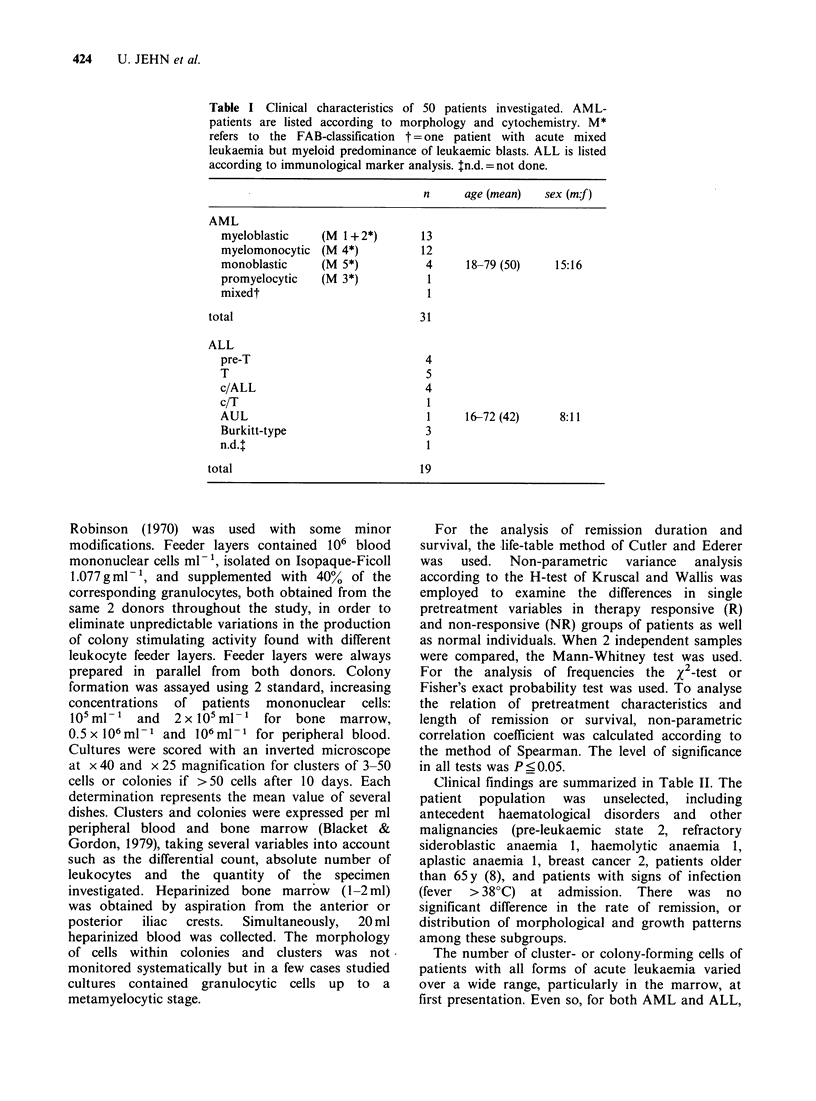

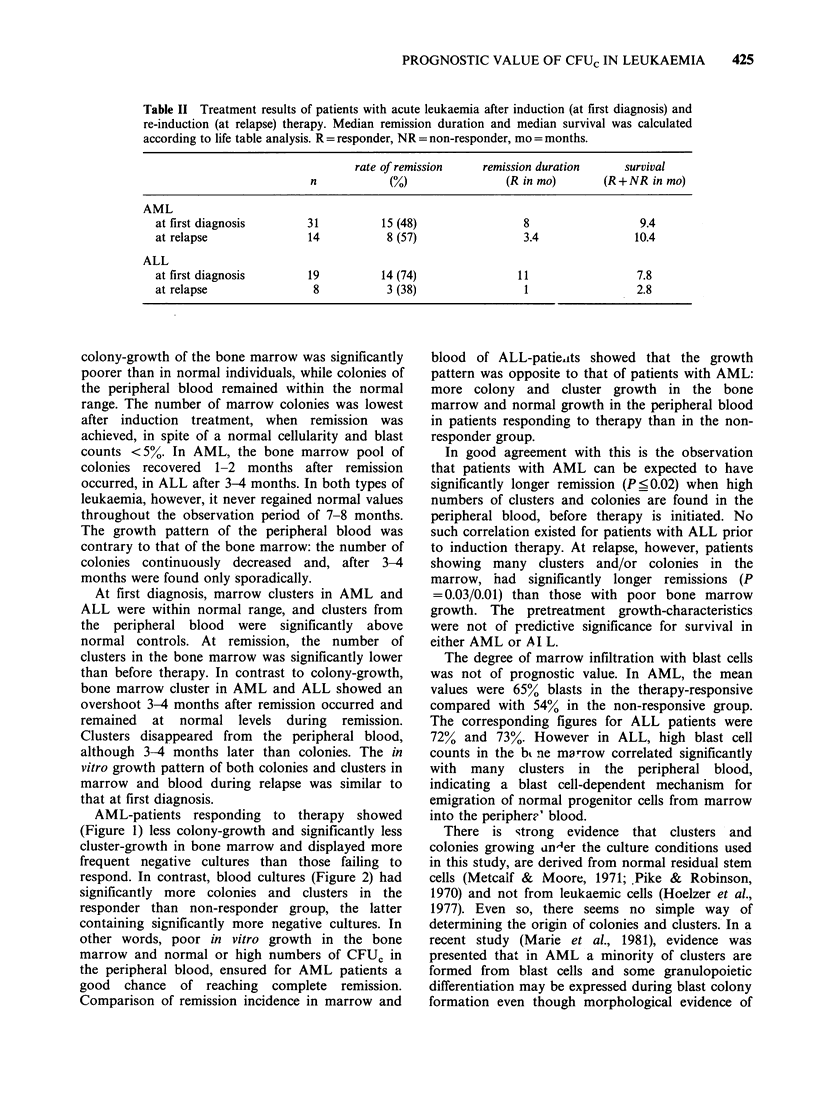

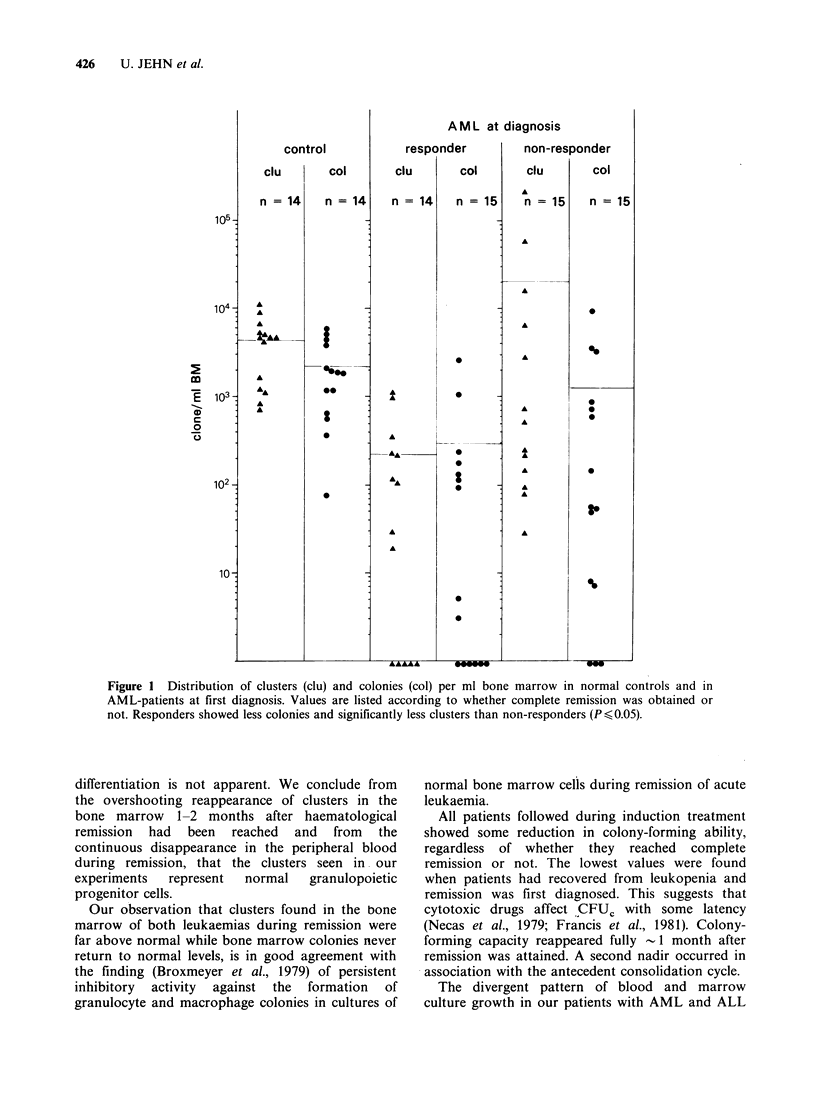

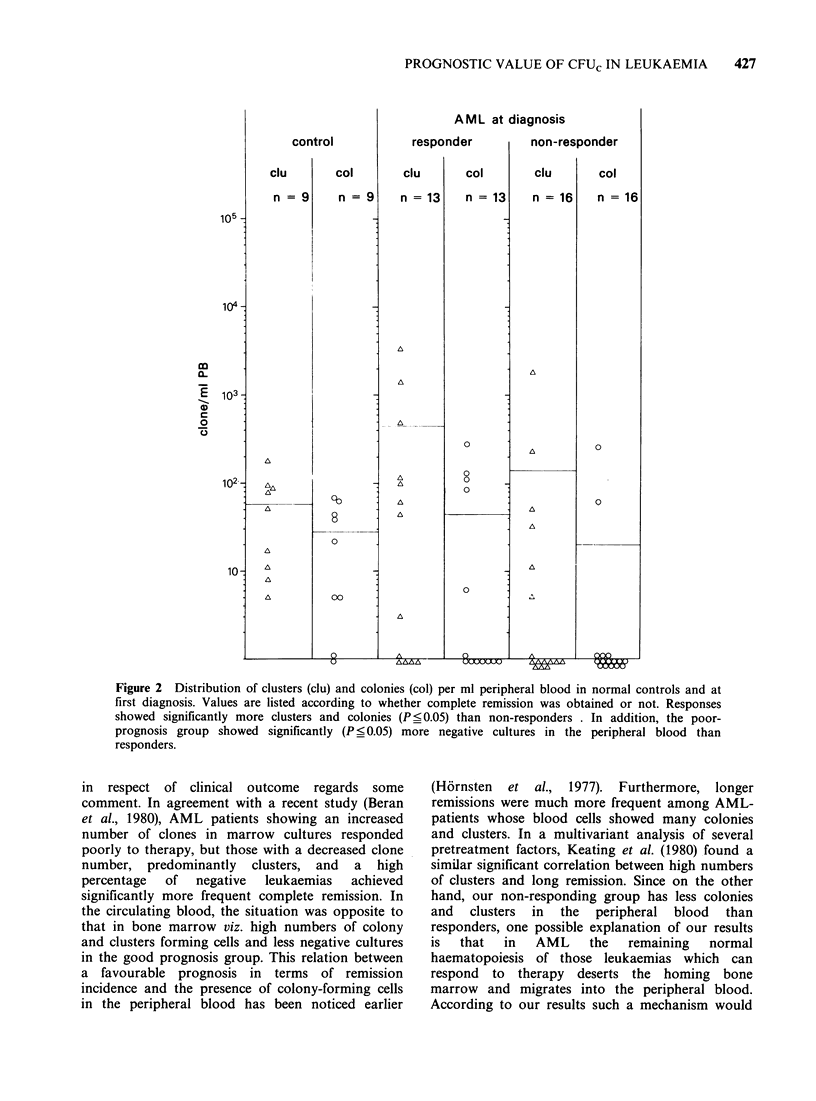

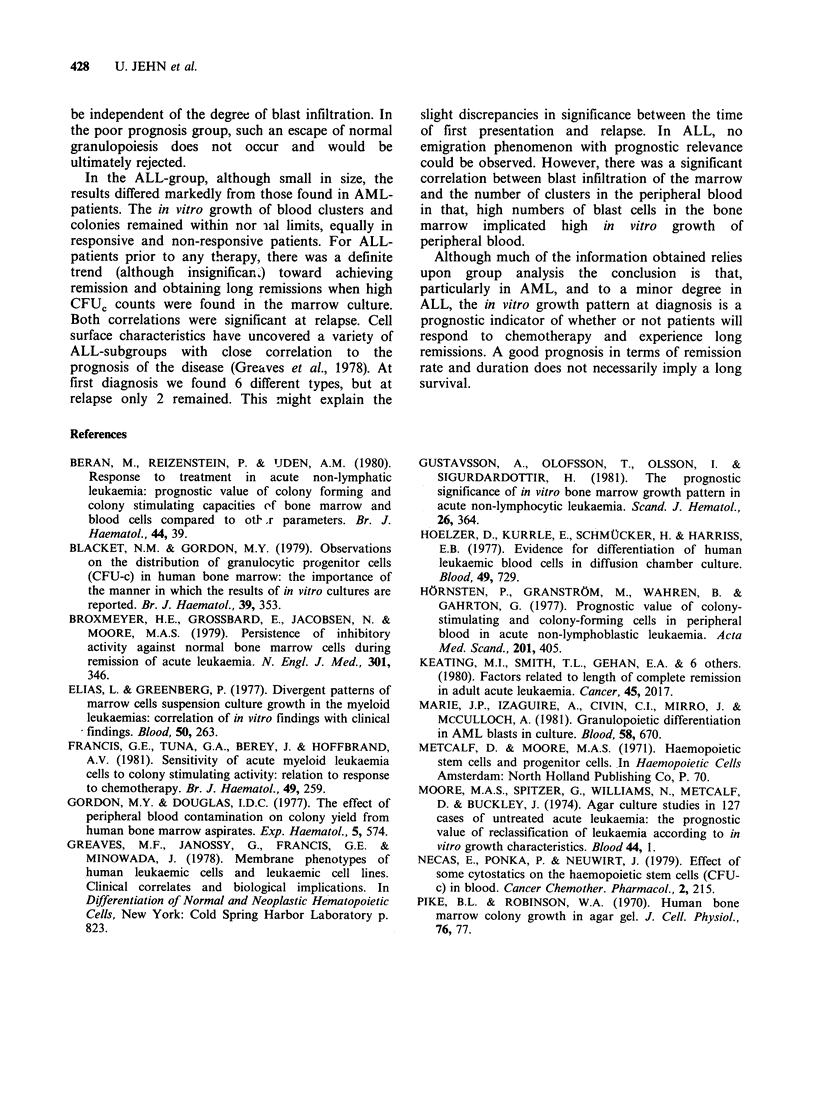


## References

[OCR_00563] Beran M., Reizenstein P., Uden A. M. (1980). Response to treatment in acute non-lymphatic leukaemia: prognostic value of colony forming and colony stimulating capacities of bone marrow and blood cells compared to other parameters.. Br J Haematol.

[OCR_00578] Broxmeyer H. E., Grossbard E., Jacobsen N., Moore M. A. (1979). Persistence of inhibitory activity against normal bone-marrow cells during remission of acute leukemia.. N Engl J Med.

[OCR_00585] Elias L., Greenberg P. (1977). Divergent patterns of marrow cell suspension culture growth in the myeloid leukemias: correlation of in vitro findings with clinical features.. Blood.

[OCR_00591] Francis G. E., Tuma G. A., Berney J. J., Hoffbrand A. V. (1981). Sensitivity of acute myeloid leukaemia cells to colony stimulating activity: relation to response to chemotherapy.. Br J Haematol.

[OCR_00610] Gustavsson A., Olofsson T., Olsson I., Sigurdardottir H. (1981). The prognostic significance of in vitro bone marrow growth pattern in acute non-lymphocytic leukaemia.. Scand J Haematol.

[OCR_00617] Hoelzer D., Kurrle E., Schmücker H., Harriss E. B. (1977). Evidence for differentiation of human leukemic blood cells in diffusion chamber culture.. Blood.

[OCR_00623] Hörnsten P., Granström M., Wahren B., Gahrton G. (1977). Prognostic value of colony-stimulating and colony-forming cells in peripheral blood in acute non-lymphoblastic leukemia.. Acta Med Scand.

[OCR_00630] Keating M. J., Smith T. L., Gehan E. A., McCredie K. B., Bodey G. P., Spitzer G., Hersh E., Gutterman J., Freireich E. J. (1980). Factors related to length of complete remission in adult acute leukemia.. Cancer.

[OCR_00635] Marie J. P., Izaguirre C. A., Civin C. I., Mirro J., McCulloch E. A. (1981). Granulopoietic differentiation in AML blasts in culture.. Blood.

[OCR_00645] Moore M. A., Spitzer G., Williams N., Metcalf D., Buckley J. (1974). Agar culture studies in 127 cases of untreated acute leukemia: the prognostic value of reclassification of leukemia according to in vitro growth characteristics.. Blood.

[OCR_00652] Necas E., Ponka P., Neuwirt J. (1979). Effect of some cytostatics on the haemopoietic stem cells (CFUs) in blood.. Cancer Chemother Pharmacol.

[OCR_00657] Pike B. L., Robinson W. A. (1970). Human bone marrow colony growth in agar-gel.. J Cell Physiol.

